# Key developments and hotspots of exosomes in Alzheimer’s disease: a bibliometric study spanning 2003 to 2023

**DOI:** 10.3389/fnagi.2024.1377672

**Published:** 2024-05-01

**Authors:** Siyu Liu, Daoying Geng

**Affiliations:** ^1^Radiology Department, Huashan Hospital, Affiliated with Fudan University, Shanghai, China; ^2^Shanghai Engineering Research Center of Intelligent Imaging for Critical Brain Diseases, Shanghai, China; ^3^Institute of Functional and Molecular Medical Imaging, Fudan University, Shanghai, China

**Keywords:** Alzheimer’s disease, exosomes, dementia, bibliometric, VOSviewer, CiteSpace

## Abstract

**Background:**

Alzheimer’s disease (AD) is a degenerative illness of the central nervous system that is irreversible and is characterized by gradual behavioral impairment and cognitive dysfunction. Researches on exosomes in AD have gradually gained the attention of scholars in recent years. However, the literatures in this research area do not yet have a comprehensive visualization analysis. The aim of this work is to use bibliometrics to identify the knowledge constructs and investigate the research frontiers and hotspots related to exosomes in AD.

**Methods:**

From January 2003 until June 2023, we searched the Web of Science Core Collection for literature on exosomes in AD. We found 585 papers total. The bibliometric study was completed using VOSviewer, the R package “bibliometrix,” and CiteSpace. The analysis covered nations, institutions, authors, journals, and keywords.

**Results:**

Following 2019, the articles on exosomes in AD increased significantly year by year. The vast majority of publications came from China and the US. The University of California System, the National Institutes of Health, and the NIH National Institute on Aging in the US were the primary research institutions. Goetzl Edward J. was frequently co-cited, while Kapogiannis Dimitrios was the most prolific author in this discipline with the greatest number of articles. Lee Mijung et al. have been prominent in the last two years in exosomes in AD. The Journal of Alzheimer’s Disease was the most widely read publication, and Alzheimers & Dementia had the highest impact factor. The Journal of Biological Chemistry, Proceedings of the National Academy of Sciences of the United States of America, and Journal of Alzheimer’s Disease were the three journals with more than 1,000 citations. The primary emphasis of this field was Alzheimer’s disease, exosomes, and extracellular vesicles; since 2017, the number of phrases pertaining to the role of exosomes in AD pathogenesis has increased annually. “Identification of preclinical Alzheimer’s disease by a profile of pathogenic proteins in neurally derived blood exosomes: a case–control study” was the reference with the greatest citing power, indicating the future steered direction in this field.

**Conclusion:**

Using bibliometrics, we have compiled the research progress and tendencies on exosomes in Alzheimer’s disease for the first time. This helps determine the objectives and paths for future study.

## Introduction

One of the most prevalent forms of dementia is Alzheimer’s disease (AD), an irreversible neurodegenerative lesion of the central nervous system with a sneaky onset (Chandra et al., 2019). The primary characteristics of AD are progressive cognitive deterioration and behavioral impairments, and it primarily affects older individuals and pre-geriatric population (Zhang X. W. et al., 2023). Memory loss and personality and behavioral changes are common clinical manifestations. In the later stage, patients gradually lose their ability to live, and eventually die of multiple complications. Amyloid β-protein (Aβ), neurogenic fibrillary tangles (NFTs), and hyperphosphorylated tau proteins aggregate abnormally to form plaques and impair neurological functioning; this is the pathological underpinning of AD (Zhou et al., 2015; He et al., 2023; Zhang X. W. et al., 2023).

Most cells discharge exosomes, which are particles with sizes ranging from 30 to 150 nm that are derived from the endosomal system and contain proteins, lipids, DNA, and other substances, into the extracellular environment (Wu et al., 2022; Chen et al., 2023). By merging with the membrane of plasma to enable endocytosis and cargo release, it modifies the microenvironment and is important for numerous processes that are both physiological and pathological (Alptekin et al., 2022). A lot of study findings on exosomes in AD have surfaced recently, covering a wide range of topics including the etiology, prognosis, and therapy. Scholars have discovered that exosomes play a role in the pathogenesis of AD by facilitating information and material movement between neurons and neuron–glia connections, which in turn influences Aβ production and accumulation via miRNAs (Ding et al., 2022; Wang Y. et al., 2022; He et al., 2023). In many literatures, exosomes have been shown to function as biomarkers and have potential value for the diagnosis of AD. Through a meta-analysis of neurological exosomes, Zhang X. et al. (2023) discovered that Aβ42, T-tau, and P-tau181 in blood neurological exosomes might be used in AD diagnosis. Gamez-Valero et al. (2019) found that distinct miRNAs in the cerebrospinal fluid of AD patients showed different modifications when in comparison to the control group, which was helpful in differentiating AD patients from healthy controls. Exosomes have received much attention as a therapeutic strategy for AD in recent years. Xie X et al. investigated the effects of allogenic human adipose mesenchymal stem cells exosomes (MSCs-exos) in patients with mild to severe AD using a three-arm, drug-intervention phase I/II clinical study. They found that intranasal administration of ahaMSCs-exos was secure and tolerated well (Xie et al., 2023). Iyaswamy et al. (2023) reported that the Fe65-engineered HT22 hippocampal neurons cell-derived exosomes induced autophagy in amyloid precursor protein (APP)-expressing neuronal cells and improved cognitive function in AD mice, confirming that it can be an effective treatment for AD.

Bibliometrics studies the distributive framework, quantitative interaction, change law, and numerical administration of texts and data using metrological approaches like mathematics and statistics. It views the literature network and bibliometric features as the research goal, and after that investigate the traits and principles of technology and science (Wu et al., 2022; Zhou et al., 2023). Keywords are analyzed for clustering and burstiness in order to investigate research patterns and hotspots. Co-citation of the references and authors captures the dynamic changes and knowledge layout of the area. VOSviewer, CiteSpace and Bibliometrix R-package are commonly used bibliometric tools. Bibliometrics is being employed in a number of medical domains, although it has not yet addressed the topic of exosomes in AD (Huang et al., 2020; Liang et al., 2023; Wang C. et al., 2023; Wang R. et al., 2023). Therefore, the purpose of this research is to obtain the knowledge structure of exosomes in AD through bibliometrics and analyzes the frontiers and hotspots, so as to lay the groundwork for the future research direction.

## Materials and methods

### Data collection method

**Figure 1:**
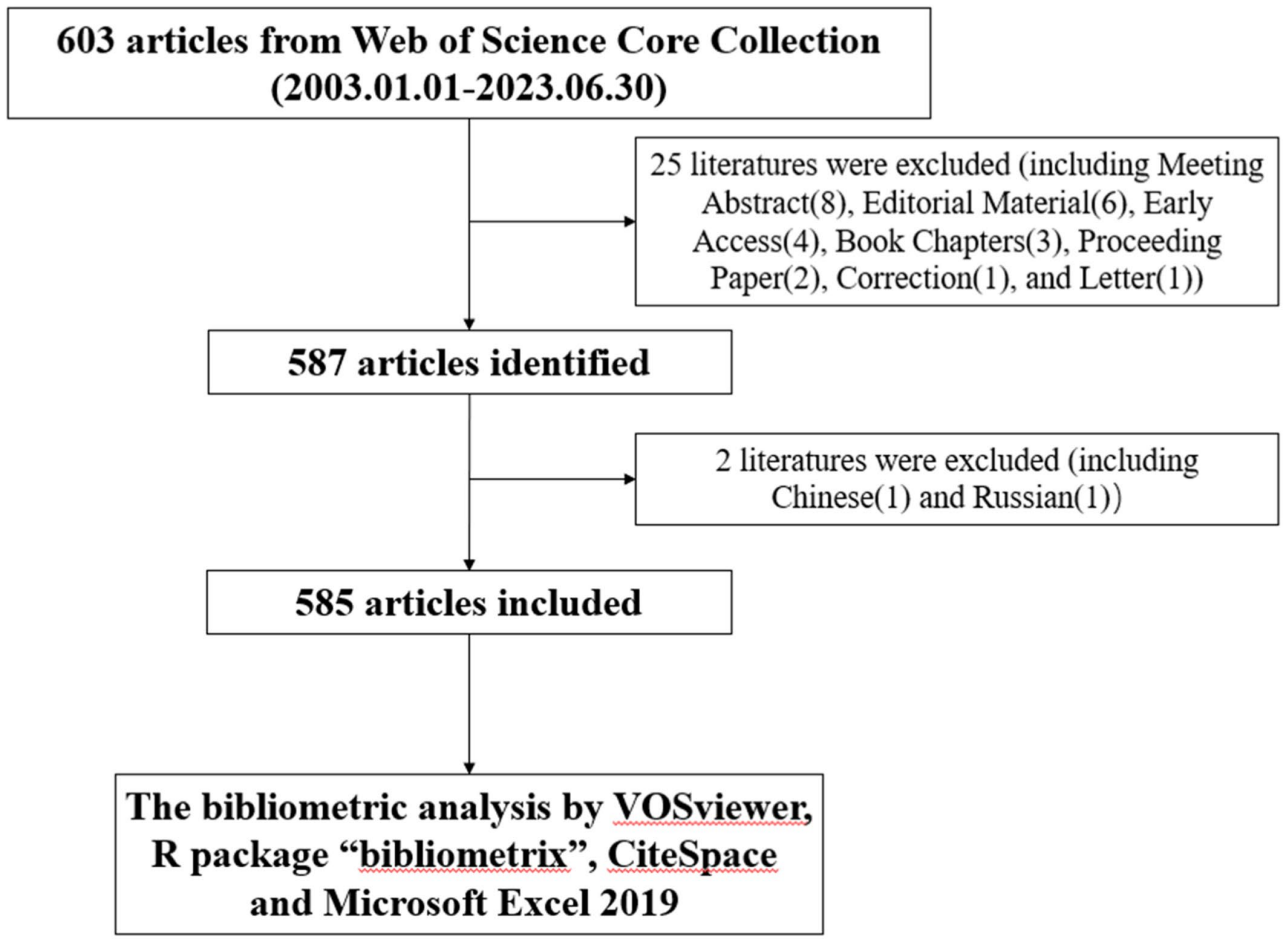
Articles retrieval flowchart.

Relevant literature searching and screening were conducted by two researchers to ensure data accuracy. In this study, web of science core collection was selected as the material origin for analysis in this study because many academics believe that Web of Science Core Collection (WOSCC) great library of digital literature resources is the best database for bibliometric studies. The search formula was [TS = (Alzheimer’s disease)] AND TS = (Exosomes) AND Languages (English) AND Document Types (Article or Review Article). The search period was 2003–2023, and data collection ended on June 30, 2023 to avoid time error. Among the information that was extracted and stored in an ordinary text document were the whole record and the cited references. Figure 1 illustrates the retrieval approach employed in this investigation. When any disagreement or problem arises, it will be resolved by negotiation and final consensus. Ultimately, 585 valid documents were included.

### Data analysis

Impact factor (IF) has always been used as a criterion for evaluating the quality of scientific journals. However, IF can no longer be used as an indicator to simply assess academic achievement with the advancements in the fields of science and technology in recent years (Garfield, 2006). Bibliometric analysis maps and visualizes the arrangement of authors, journals, references, nations, and other data to provide insight into the state of research and innovation in a certain topic (Roldan-Valadez et al., 2019). To determine these trend distributions, we used VOSviewer (version 1.6.20), CiteSpace (version 6.2.R4), the Bibliometrix package from R (version X64 4.3.1) and Microsoft Excel 2019.

A software program called VOSviewer^1^ is used to create maps based on web data and to visualize and navigate these maps (Van Eck and Waltman, 2010). The program was used in our study to analyze the following: nation, institution, author, and co-cited author; journal, and co-cited journal; keyword; and co-cited reference using network visualization, overlay visualization, and/or density visualization. The total amount of projects is represented by the dimension of the nodes on the map, and the level of cooperation is indicated by the width of the lines connecting the nodes (Wu et al., 2022).

The R package “bibliometrix”^2^ is an R-based bibliometrics program that requires the download of R and Rstudio (Zhou et al., 2023). This software was applied to know country scientific production, most cited journals, most frequent keywords, trend topics and most cited references.

CiteSpace^3^ is a bibliographic presentation and evaluation software that is aimed at examining the potential knowledge presenting in scientific analyzes and is progressively developed within the overall structure of scientometrics and data visualization (Synnestvedt et al., 2005). In this work, we employed CiteSpace to calculate the quantity of national, institutional, and author publications; create a dual-map overlay of journals; and examine cited authors, keywords, and references that exhibited the strongest bursts of citations.

Furthermore, the yearly number of posts exported by CiteSpace was examined using Microsoft Excel 2019.

## Results

### Publication and growth trend

**Figure 2:**
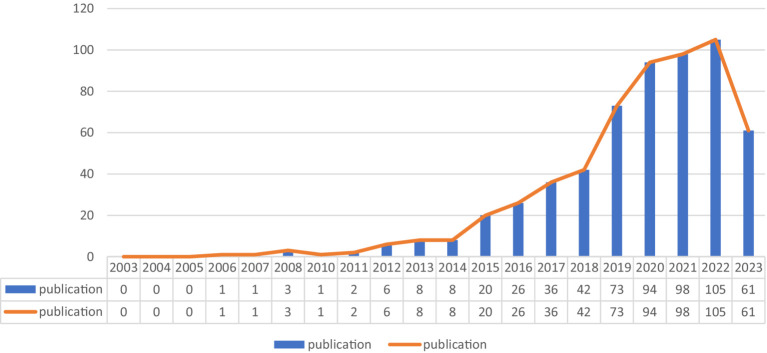
Average annual publications on exosome in AD from 2003 to 2023.

As is shown in Figure 2, upward trend in the annual publications of exosomes in AD issued per year. The number of publications between 2003–2005 was zero, meaning that no AD exosome-related studies were conducted during this period. The researches of exosomes in AD began to appear from 2006 to 2018, with annual publication volume increasing each year. The average number of articles per year for this period was 12. While, the number of papers showed a significant increase from 2019 to 2023, and the growth rate was also obviously faster, with an average of 86 publications per year.

### Country and institutional distribution analysis

**Figure 3:**
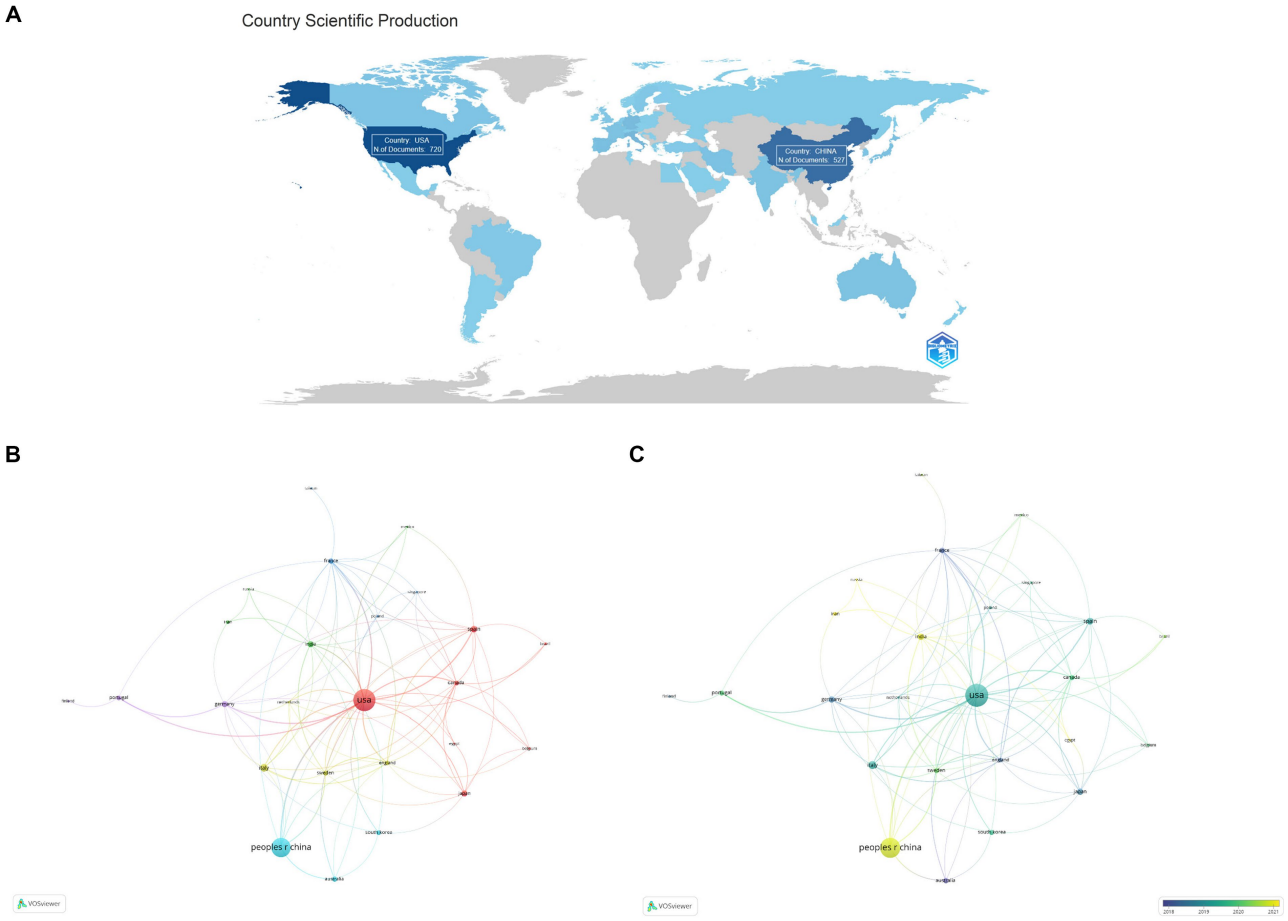
Country publication analysis. **(A)** World map of national documentation outputs. **(B)** Network visualization of cooperative relationships between multiple countries. **(C)** Overlay visualization of cooperative relationships between multiple countries.

The 585 articles in this study came from 51 countries, and 266 institutions conducted studies in this topic. The top 10 nations when it came to the overall amounts of articles were USA (196), China (158), Italy (40), Spain (30), India (28), Japan (28), Germany (27), Australia (23), France (22) and Canada (21) in the last two decades, with a high concentration of countries in Asia, North America and Europe (Table 1). The two countries with the most papers were the United States and China, both of which also had high centrality, at 0.9 and 0.04, respectively (Figure 3A). Then we constructed collaborative network for the top 25 countries, with nodes representing countries, and found that various nations were actively cooperating with one another (Figures 3B,C).

**Table 1:** The top 10 countries and institutions on articles of exosomes in AD.

Rank	Country	Counts	Institution	Country	Counts
1	United States	196	University of California System	America	48
2	PR China	158	National Institutes of Health (NIH) – USA	America	35
3	Italy	40	NIH National Institute on Aging (NIA)	America	35
4	Spain	30	University of California San Francisco	America	23
5	India	28	US Department of Veterans Affairs	America	17
6	Japan	28	Harvard University	America	16
7	Germany	27	Veterans Health Administration (VHA)	America	15
8	Australia	23	Boston University	America	15
9	France	22	Capital Medical University	China	14
10	Canada	21	Shanghai Jiao Tong University	China	13

**Figure 4:**
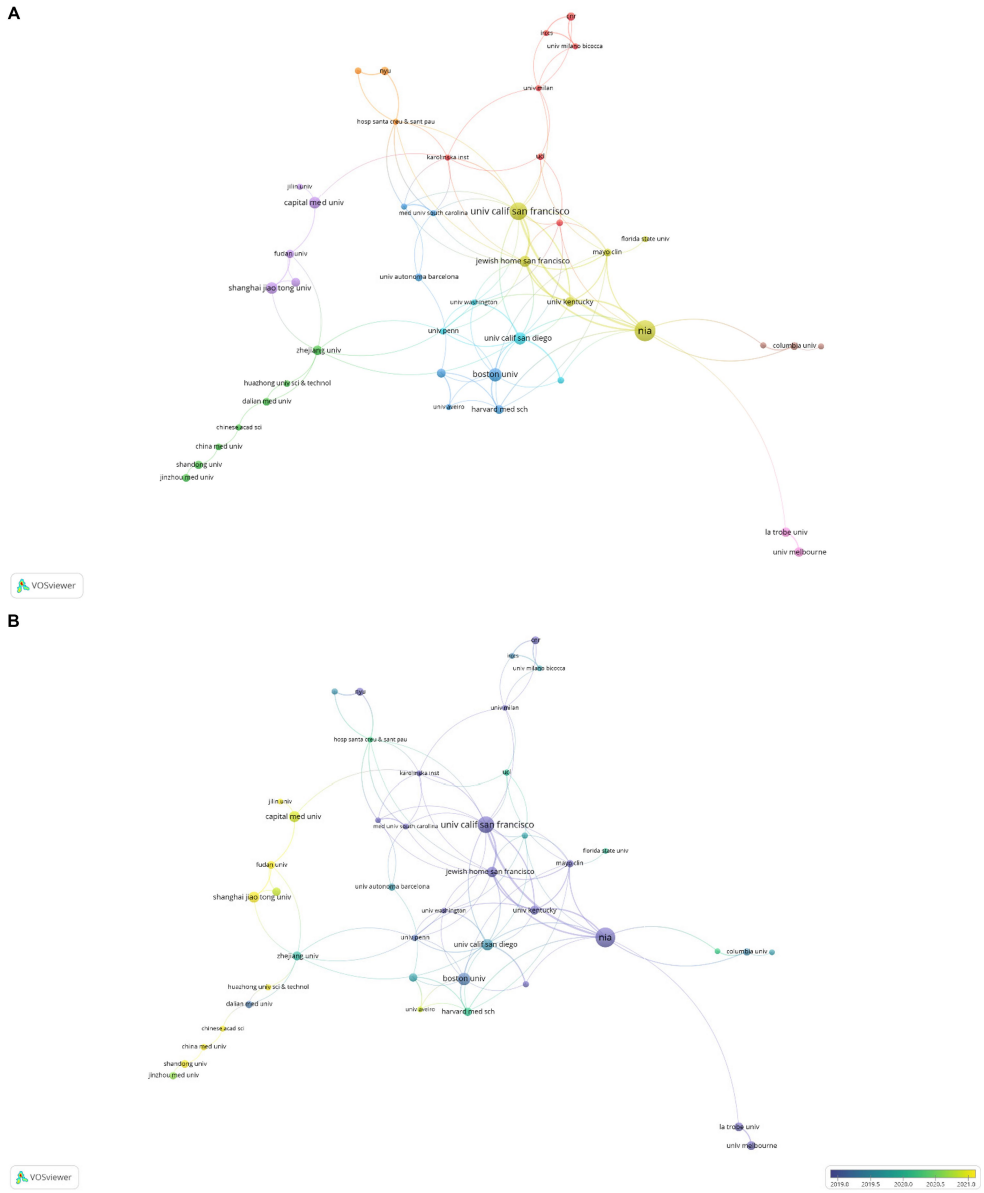
Institution publication analysis. **(A)** Network visualization of collaborative links between different institutions. **(B)** Overlay visualization of collaborative links between different institutions.

The top 10 institutions were spread across China and the US in accordance with the volume of national publications; the only Chinese universities in the top 10 were Capital Medical University and Shanghai Jiao Tong University (Table 1). The centralities of Shanghai Jiao Tong University and Capital Medical University were slightly inferior to the other top 8 US institutions with only 0.01 and 0.02. Similarly, we constructed relationship network for the top 44 institutions, nine of which occupied cooperative clustering centers, namely Consiglio Nazionale delle Ricerche (CNR), China Medical University, Boston University, Florida State University, Capital Medical University, University of California San Diego, Hospital of Santa Creu i Sant Pau, Columbia University and La Trobe University (Figure 4).

### Author contributions and co-cited authors

**Figure 5:**
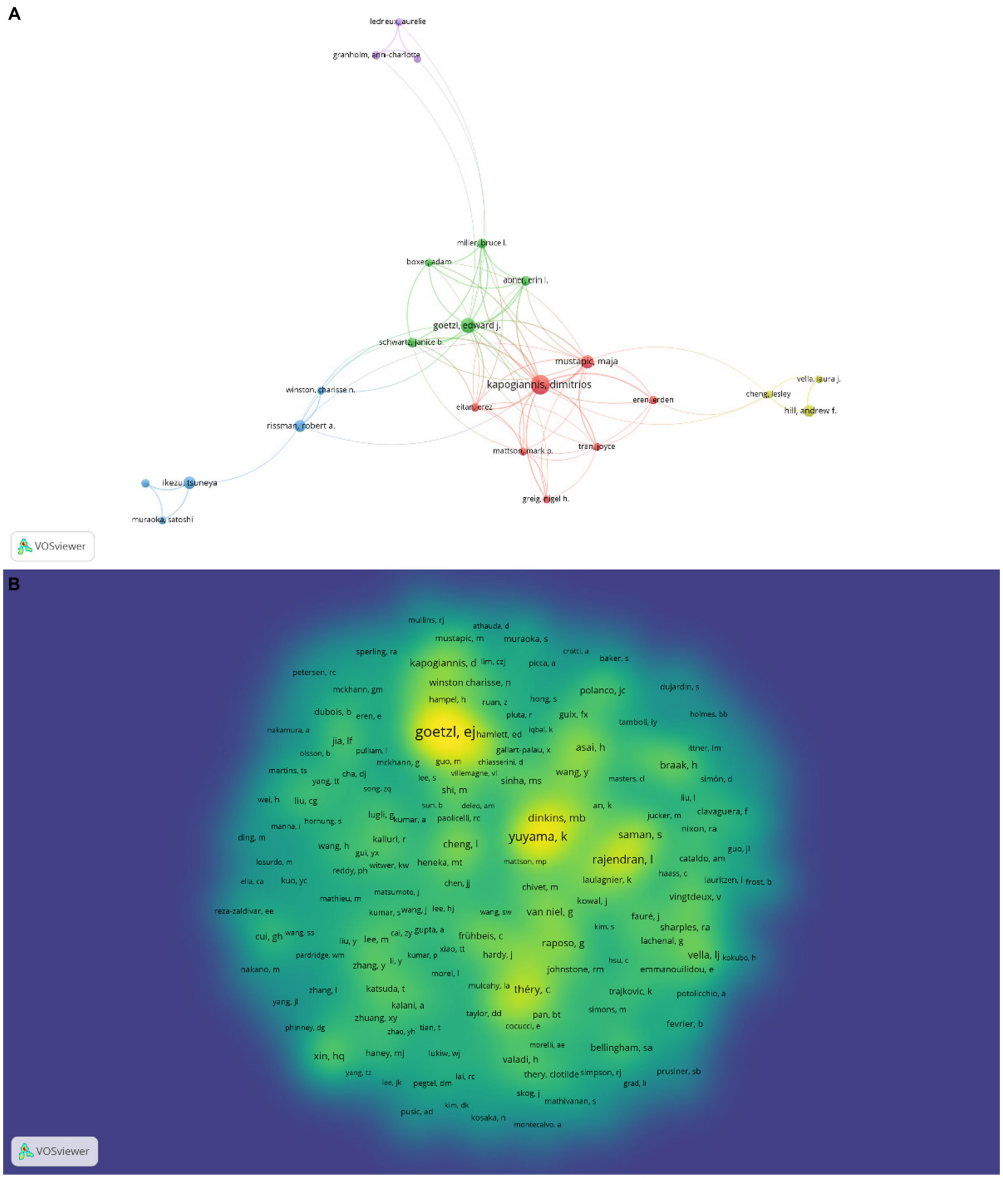
Author and co-cited authors analysis. **(A)** Network visualization of the relationships of different authors. **(B)** Density visualization of the relationships of co-cited authors.

In this discipline, 278 scholars have achieved success and released works in the last 20 years, and we identify the top 10 authors based on the quantity of papers produced (Table 2). Among the highly productive authors, Kapogiannis Dimitrios was the No.1 with 32 articles, followed by Goetzl Edward J (20) and Mustapic Maja (14). In addition, we used VOSviewer to find close interaction and collaboration between multiple authors, such as Kapogiannis Dimitrios and Mustapic Maja, Goetzl Edward J and Miller Bruce L and Rissman Robert A and Winston Charisse (Figure 5). One noteworthy point in the author analysis was that two of the top 10 authors came from China whose number of publications also ranked high, respectively from Capital Medical University and Fudan University.

**Table 2:** The top 10 most prolific authors.

Author	Count	Centrality	Year
Kapogiannis, Dimitrios	30	0.03	2015
Goetzl, Edward J.	19	0.01	2015
Mustapic, Maja	11	0.01	2017
Ikezu, Tsuneya	9	0	2019
Rissman, Robert A.	6	0.02	2017
Abner, Erin L.	6	0	2015
Hill, Andrew F.	6	0.02	2008
Miller, Bruce L.	5	0	2015
Levy, Efrat	5	0	2011
Ikezu, Seiko	5	0	2020

**Figure 6:**
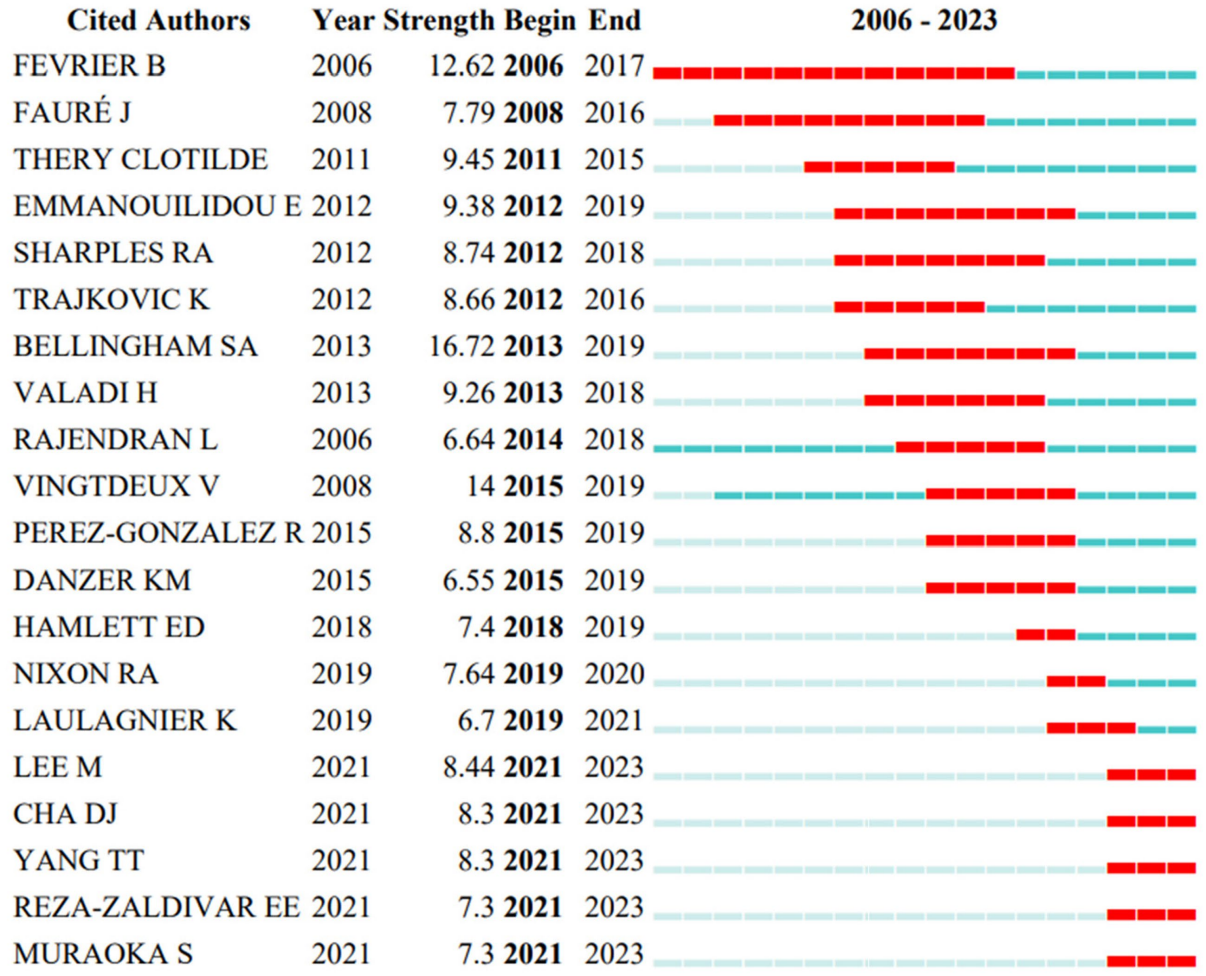
Top 20 cited authors with the strongest citation bursts.

Goetzl Edward J (476) was the most co-cited author, followed by Yuyama Kohei (332) and Rajendran Lawrence (213). Through their co-cited author relationship network, it was discovered that co-cited authors also worked closely together (Figure 5). We discovered that the number of citations for Lee Mijung, Cha Diana J, Yang Ting Ting, Reza-Zaldivar Edwin E and Muraoka Satoshi had increased significantly over the past two years by using CiteSpace to carry out burstness detection, suggesting that these researchers had more outstanding performance in this area of study (Figure 6).

### Journal analysis and co-cited journals

There were 256 journals with articles on exosomes in AD; the Journal of Alzheimer’s Disease had the highest frequency of publications (5.5%), followed by the International Journal of Molecular Sciences (3.9%), Cells (3.1%), Frontiers in Aging Neuroscience (2.9%), and Frontiers in Neuroscience (2.4%). Journal Citation Reports (JCR) classified half of the top 10 journals’ publications as Q1 and the other half as Q2. Alzheimers & Dementia had the highest impact factor in Q1 (IF = 14.0) (Table 3).

**Table 3:** The top 10 most productive journals and co-cited journals on articles of exosomes in AD.

Rank	Journal	Count	JCR	IF	Co-cited Journal	Count	JCR	IF
1	Journal of Alzheimer disease	32	Q2	4.0	Journal of Biological Chemistry	1893	Q2	4.8
2	International Journal of Molecular Sciences	23	Q1	5.6	Proceedings of the National Academy of Sciences of the USA	1,166	Q1	11.1
3	Cells	18	Q2	6.0	Journal of Alzheimer’s disease	1,058	Q2	4.0
4	Frontiers in Aging Neuroscience	17	Q2	4.8	Journal of neuroscience	937	Q1	5.3
5	Frontiers in Neuroscience	14	Q2	4.3	PloS one	899	Q2	3.7
6	Alzheimers & Dementia	12	Q1	14.0	Alzheimer’s & Dementia	866	Q1	14
7	FASEB Journal	12	Q1	4.8	Nature	704	Q1	64.8
8	Molecular Neurobiology	11	Q2	5.1	Scientific Reports -UK	694	Q2	4.6
9	Biomedicines	9	Q1	4.7	FASEB Journal	690	Q2	4.8
10	Frontiers in Cell and Developmental Biology	9	Q1	5.5	Journal of Extracellular Vesicles	658	Q1	16

Journal of Biological Chemistry (Q2, IF = 4.8) had the most citations among the top 10 co-cited journals, with three of them receiving more than 1,000 citations. Proceedings of the National Academy of Sciences of the United States of America (Q1, IF = 11.1) and Journal of Alzheimer’s Disease (Q2, IF = 4.0) were the next most cited journals (Table 3).

**Figure 7:**
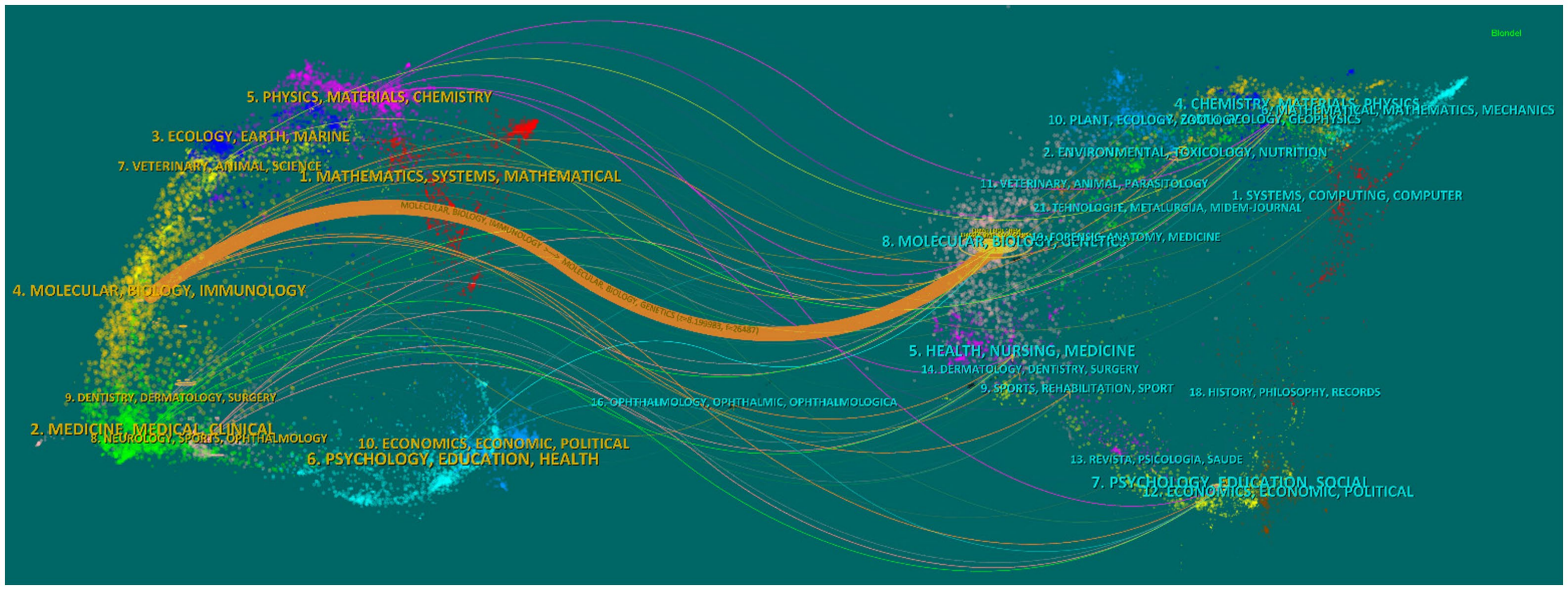
The dual-map overlay of journals on articles of exosomes in AD.

The dual-map overlay of journals on exosomes in AD represented the citation relationship between the citing journals on the left section and the cited journals on the right in Figure 7. For each citation path, the thickest orange line indicated the most important one. Molecular, Biology, and Genetics tended to have an influence on articles published in Molecular, Biology, and Immunology (Figure 7).

### Keyword analysis and hotspots

**Figure 8:**
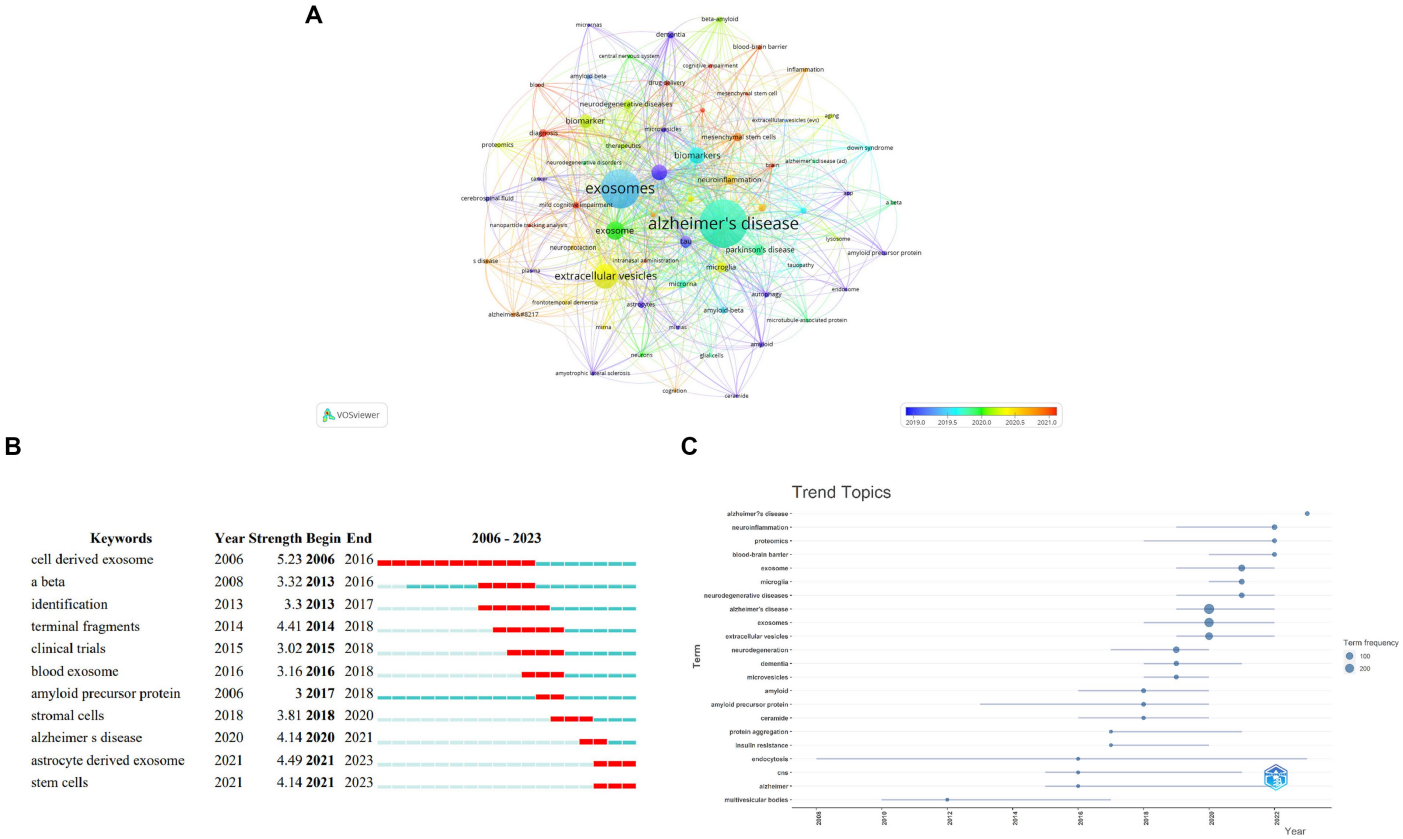
Keyword analysis. **(A)** Overlay visualization of keyword clusters. **(B)** Top 11 keywords with the strongest citation bursts and **(C)** trend topics analysis on publications of exosomes in AD.

Keywords indicate The core and theme of a field of study. In order To explore The research hotspots about exosomes In AD In The last 20 years, We carried Out co-word analysis and clustering. Among 1,271 keywords involved In 585 articles, The most frequent keyword was Alzheimer’s disease (298), followed By exosomes (225) and extracellular vesicles (112) represented The main direction of this topic. In Table 4, The Top 10 keywords from author keywords were displayed (Table 4). We included The titles and abstracts In The keyword analysis and again obtained similar results. We constructed The keyword network and created 6 clusters of different colors and sizes By VOSviewer with marked overlaps demonstrating strong linkages between clusters (Figure 8). The keywords of cluster 1 included blood–brain barrier, cognitive impairment, drug delivery and neuroprotection. Cluster 2 Was mainly composed of keywords like biomarkers, dementia and plasma. Cluster 3 consisted mostly of The pathophysiologic products of AD such As amyloid beta and tau protein. The keywords In cluster 4, 5 and 6 were fewer than that In The formers including microvesicles, extracellular vesicle, lysosome and So On.

**Table 4:** The top 10 most frequent keywords from author keywords.

Words	Occurrences
Alzheimer’s disease	298
Exosomes	225
Extracellular vesicles	112
Exosome	72
Biomarkers	60
Neurodegeneration	59
Biomarker	44
Tau	37
Parkinson’s disease	34
Microglia	32

The top 11 keywords with the strongest citation bursts in Figure 8 showed the burst time periods of different keywords by a red line. Cell derived exosome had the greatest bursting intensity (5.23) followed by astrocyte derived exosome (4.49) and terminal fragments (4.41) from 2006 to 2023. It’s worth noting that keywords like astrocyte derived exosome and stem cells had only been around in 2023 (Figure 8). The trend topic analysis of keywords indicated that most studies focused on endocytosis and multivesicular bodies before 2016. While since 2017 the researchers have begun to explore role of exosomes in AD pathogenesis because the term frequency of these keywords has gradually increased over the years such as extracellular vesicles microglia neuroinflammation microvesicles and proteomics which implies the future hotspots of exosomes in AD (Figure 8).

### Co-cited references and burst references analysis

**Figure 9:**
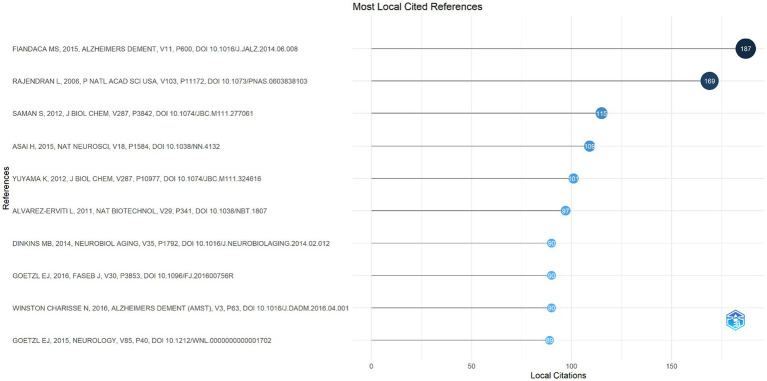
The top 10 most co-cited references.

**Figure 10:**
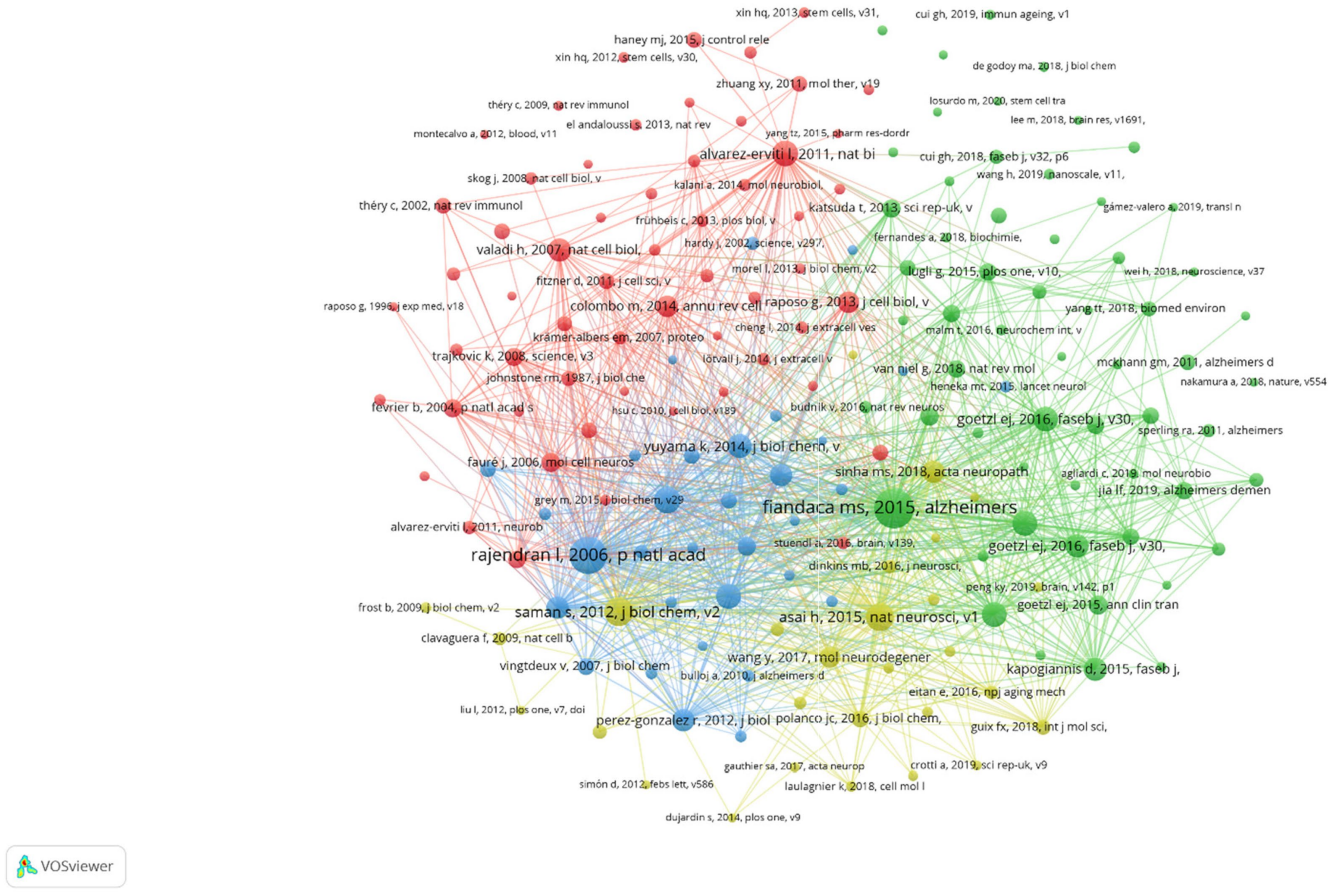
The network visualization of the relationships of co-cited references.

Finding high-impact publications and examining the frontier and future directions of this subject are the goals of article co-citation analysis. Based on the analysis of R package “bibliometrix,” there were 30,299 cited references in the 585 articles, and among these cited references, top 5 documents were cited more than 100 times (Figure 9). In order to create a visual representation of the co-citation relationship network, we used VOSviewer to analyze 176 items overall, with 20 citations required for a reference to be considered referenced. Nodes were linked to one another by straight lines, and each node represented an article. According to Figure 10, 176 cited references were separated into 4 colored clusters (Figure 10). The core of cluster 1 (red) was “Alvarez-Erviti L, 2011, Nat Biotechnol, V29, P341, DOI 10.1038/NBT.1807,” which demonstrated the curative benefits of exosome-mediated siRNA delivery. Cluster 2, represented in green, consisted of 58 articles focusing on the discovery of multiple proteins of exosomes in AD. The two most highly cited documents in cluster 3 (blue) and cluster 4 (yellow) are “Rajendran L, 2006, Proc Natl Acad Sci USA, V103, P11172, DOI 10.1073/PNAS.0603838103” and “Saman S, 2012, J Biol Chem, V287, P3842, DOI 10.1074/JBC.M111.277061.” Meanwhile, these literatures also show a rich co-citation relationship with each other.

**Figure 11:**
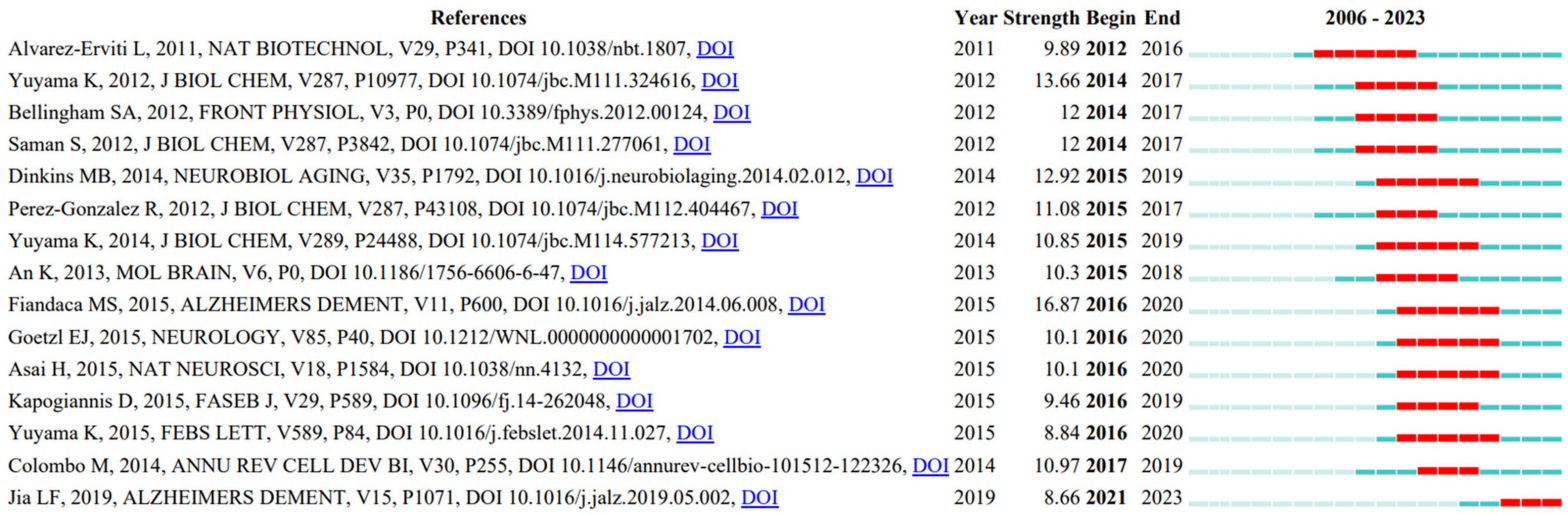
Top 15 references with the strongest citation bursts.

The top 15 references in our analysis, which represented commonly referenced sources by academics in this subject between 2006 and 2023, had the strongest citation spikes. The reference with the highest citation strength (16.87) was “Identification of preclinical Alzheimer’s disease by a profile of pathogenic proteins in neurally derived blood exosomes: A case–control study” published by Fiandaca et al. (2015) (Figure 11). This paper, which had a spike in citations from 2016 to 2020 and directed research direction, discovered that levels of P-S396-tau, P-T181-tau, and Aβ1-42 in extracts of neurally generated blood exosomes might anticipate the progression of AD as long as 10 years before clinical onset.

## Discussion

In this research, we analyzed 585 articles and review them from WOSCC over the period 2003–2023 from multiple perspectives by bibliometrics and found that the study and development of exosomes in AD has quickly expanded in the past few decades. More and more scholars at home and abroad have sought breakthroughs and obtained results in cell derived exosome, microglia, proteomics and other directions.

Generally speaking, no researchers had explored the relationship between exosomes and AD prior to 2006. Between 2006 and 2018, the average number of annual publications was 12, while from 2019 to 2023, the average annual volume of articles has grown rapidly to 86. This result suggested that a significant number of researchers had started concentrating on pertinent studies. Three major nations published exosomes in AD: the USA, China, and Italy, with the USA ranking highest. The top institution, University of California System, is also from the USA, meaning that the USA is ahead of other countries and regions in research progress in this field. In comparison, Capital Medical University and Shanghai Jiao Tong University from China ranked ninth and tenth, respectively, among the top 10 institutions, indicating that so far Chinese institutions had been conducting research in exosomes of AD, but still needed to make efforts. In terms of the country and institution cooperative relationship network, the close relationship between the USA and China is also one of the reasons why these two countries have the highest volume of articles published. Therefore, we believe that extensive cooperation and exchanges among institutions of various countries are conducive to the development of exosomes in AD.

Based on author analysis, Kapogiannis Dimitrios (USA), Goetzl Edward J (USA) and Mustapic Maja (USA) are the most productive authors in this area, and Goetzl Edward J (USA) is also the most frequently co-cited author. All of them have made tremendous contributions to the research on exosomes in AD. These three authors have collaborated since 2015 and have co-published 6 articles on exosomes in AD so far, pioneering the study of this area. Their reports, which centered on the role of exosomes in the pathogenesis of AD, noted that patients with white matter hyperintensity may have small cerebral vascular disease manifest at the preclinical or MCI stage due to high endothelial-derived exosome levels of Aβ40, Aβ42, and phosphor-181 T-tau. Low exosomal levels of survival proteins may also result in decreased resistance to AD neurotoxic proteins (Goetzl et al., 2015; Abner et al., 2020). They also investigated the application of exosomes in the management of AD, one of which indicated that astrocyte-derived exosome cargo proteins could contribute to study the impact of β-site amyloid precursor protein-cleaving enzyme 1 inhibitors in AD (Goetzl et al., 2016).

Researchers who are interested in exosome in AD should pay more attention to the top 10 journals and cited journals, such as Journal of Alzheimer’s Disease, Frontiers in Aging Neuroscience and Alzheimers & Dementia, because these journals are more influential and allow researchers to delve deeper into this field.

Exact keyword analysis is crucial for identifying research hotspots and guiding future developments in a specific topic. High-frequency keywords are mainly related to AD (Alzheimer’s disease, neurodegeneration and tau) and exosomes (exosomes, extracellular vesicles, biomarkers and microglia), which are also our research directions. While, the top keywords with the strongest citation bursts and the trend topic analysis of keywords provide a clear indication of dynamic changes and trends in exosomes in AD. Beginning in 2017, researchers discovered the role of exosomes in the pathogenesis of AD, with cell-derived exosomes, astrocyte-derived exosomes, and stem cells becoming the main subjects of study. Since the pathologic basis of AD is a neurodegenerative illness marked by tau and Aβ deposition, the majority of authors have chosen to investigate the connection between exosome-related terms and these two products.

When it comes to biomarkers, RNA is mentioned. Numerous RNAs can be employed as biomarkers for the diagnosis, management, and prognosis of AD. Wang Z. Y. et al. (2022) summarized various functions of exosomal noncoding RNAs in AD in a review published in Frontiers in molecular neuroscience in 2022 and found that different RNAs have different roles in affecting the elimination and accumulation of Aβ, APP and tau, which can be used as good pathological modulators and biomarkers. MiRNA has the largest number of non-coding RNA subtypes. Many scholars have found that the target of miRNA is APP, which can promote APP expression and Aβ deposition and affect energy metabolism to advance the development of AD (Liu et al., 2014; Sarkar et al., 2016; Chen et al., 2019).

The exosome produced by microglia is secreted by exocytosis, which is rich in receptors and kinases and is essential to cell signal transduction. For instance, Asai et al. (2015) found that microglia produce tau-containing exosomes, which can be ingested by neurons and raise the amount of tau protein. Joshi et al. (2014) suggested that microglia-derived microvesicles can increase the neurotoxicity of Aβ, which is a new mechanism for microglia involvement in AD degeneration. Tau and Aβ can be delivered to the brain by microglia-derived exosomes, and microglia can also absorb and eliminate them for therapeutic reasons. According to reports, Neuro2a cell-derived exosomes have the ability to transform extracellular Aβ into amyloid fibers and facilitate Aβ uptake by microglia, thereby lowering its concentration and mitigating synaptic toxicity in the hippocampus (Chang et al., 2013; Yuyama et al., 2014).

Researchers have long viewed stem cells as the cutting edge of regenerative medicine. Stem cell-generated exosomes, like exosomes derived from other cells, transport a range of RNA, proteins, and lipids that are important for disease diagnosis and treatment. In the researches of AD, there are most studies on exosomes derived from neural stem cells (NSCs), MSCs and adipose-derived stem cells (ADSCs) (Lee et al., 2018; Natale et al., 2022). Li et al. (2020) demonstrated that NSCs-exo ameliorated cognitive dysfunction of AD model mice, reduced inflammatory response, and affected the AD pathological environment to some extent. Lee et al. (2018) utilized the NSCs of Tg2576 AD mice to study the effect of ADSCs-exo on AD phenotypes to explore the therapeutic ability of ADSCs-exo in AD, and indicated that ADSCs-exo reduced the levels of AB42 and Ab40 and AB42/40 ratio in AD cells, and decreased the rate of apoptosis in AD neurons, confirming that ADSCs-exo can be part of a therapeutic strategy. It is evident that the role of cell-derived exosomes in the pathogenesis and treatment of AD has become more and more prominent in the last five years. Related *in vivo* and *in vitro* experiments are also increasing year by year. In future research and experiments, we can refer to these mature cells to explore the mechanism at a deeper level, or find new cells to use similar experimental methods to obtain new research results.

A co-citation is a reference that has been mentioned by several other articles and represents the foundation for the research that scientists working in a particular area need to draw upon in their preliminary explorations. As is shown in Figure 9, we displayed the top 10 co-cited references. With 187 citations overall, the most referenced paper was released in 2015 by Fiandaca et al. (2015) and this study demonstrated that levels of different tau proteins in neurogenic blood exosome extracts were related to the future development of AD to some extent, which set the stage for the application of exosomes in AD. This article is also the core of cluster 2 and the reference with the highest citation strength in the top 15 references with the strongest citation bursts. Winston et al. (2016) also reached similar conclusions as Fiandaca et al. in predicting AD conversion. The study on AD beta-amyloid peptides published by Rajendran et al. in Proceedings of the National Academy of Sciences of the United States of America in 2016 is the second most frequently referenced source and the earliest of these 10 articles. They found that exosomal proteins accumulate in brain plaques of AD patients, suggesting that exosomes also play a role in the pathogenesis of AD, which advances our understanding of the disease’s etiology (Rajendran et al., 2006). Likewise, Saman et al. also made achievements in the pathogenesis of AD. They showed that exosome-mediated phosphorylation of tau proteins plays a role in the elevation of cerebrospinal fluid tau protein in the early stages of AD, demonstrating a positive correlation between exosomes and tau protein levels (Saman et al., 2012). After qualitatively analyzing the most cited literature, we found that the role of exosomes in AD is more directed towards pathogenesis, which also guides the subsequent direction of researchers. Besides, 4 articles were about exosome-related AD therapy among the top 10 co-cited literature. Several scholars have indicated that some drugs and pathways can significantly reduce the level of tau protein *in vivo* by inhibiting the synthesis and secretion of exosomes by microglia, and siRNA is often involved in this process (Alvarez-Erviti et al., 2011; Yuyama et al., 2012; Dinkins et al., 2014; Asai et al., 2015). In summary, these publications provide information on the use of exosomes in the etiology, management, and prognosis of AD, which is helpful for researchers to gain an initial understanding of the field.

In this work, we organized the present research status and summarized the research hotspots of exosomes in AD using CiteSpace, the R package “bibliometrix,” and VOSviewer. Bibliometric analysis is easy to understand and operate. We can rapidly and intuitively understand the overall state of research and the direction of a field’s development, and identify articles and journals that have received more attention in the field using bibliometric knowledge. However, the current study has many shortcomings and restrictions. Firstly, the articles and review articles collected in this research are only from WOSCC and other databases are ignored such as PubMed and Scopus, which will lead to incomplete analysis of the data and omission of some relevant studies. Next, only English publications are included in this research, excluding articles in other language. Thirdly, only the author’s keywords are analyzed in keyword analysis, which means that some articles without author’s keywords may be left out.

## Conclusion

In this study, we used bibliometrics to explore research hotspots of exosomes and AD for the first time with VOSviewer, R package “bibliometrix” and CiteSpace software. Following 2019, there have been an increasing number of released research papers on relevant content annually, suggesting that many academics are interested in the study of exosomes in AD. The development of this discipline has been led by the US and China, although Chinese researchers’ and institutions’ research accomplishments need to be strengthened. Journal of Alzheimer’s Disease and Journal of Biological Chemistry are important journals that deserve to be noted and referenced. Keywords like neuroinflammation, microvesicles, and proteomics are frequently used, indicating that further research into the function of exosomes in the pathophysiology of AD is warranted. The use of bibliometrics allows for the creation of knowledge domains and the prediction of emerging trends, providing researchers with more definite research goals and directions.

## Data availability statement

The original contributions presented in the study are included in the article/supplementary material, further inquiries can be directed to the corresponding author.

## Author contributions

SL: Writing – original draft. DG: Writing – review & editing.

## Footnotes

^1^ www.vosviewer.com

^2^ https://www.bibliometrix.org

^3^ http://cluster.ischool.drexel.edu
